# Epidemiology of Infantile Visceral Leishmaniasis in Western Algerian And The Convenience of Serum For The Disease Diagnosis by PCR and Immunochromatography

**DOI:** 10.22088/IJMCM.BUMS.7.1.32

**Published:** 2018-03-14

**Authors:** Hadj Slimane Touria, Senouci Kheira, Midoun Nori, Bouchetara Assia, Laradj Amel, Bittar Fadi

**Affiliations:** 1 *Natural and Life Sciences Faculty* *, Department of Biology, University of Oran 1 Ahmed Ben Bella, Oran, Algeria.*; 2 *Department of Epidemiology and Preventive Medicine, * *University Hospital of Oran (EHU)* *, Oran, Algeria.*; 3 *Infectious Diseases Department, Pediatric Hospital of Oran (EHS)* *, Oran, Algeria.*; 4 *Aix-Marseille University- Faculty of Pharmacy, IHU-Méditerranée Infection, Marseille, France.*

**Keywords:** Imunochromatography, RT-PCR, bone marrow, serum, sequencing, Leishmania infantum

## Abstract

Epidemiological situation of infantile visceral leishmaniasis (IVL), which is a public health problem in Algeria, is almost unknown in the cities of Western part of the country. The aim of this study was to analyze the epidemiological, clinical, biological, therapeutic, and evolutionary aspects of IVL in Western Algeria, to evaluate the performance of the immunochromatography as a rapid diagnostic test of the disease, and to propose a diagnosis approach by real-time polymerase chain reaction (RT-PCR) assay from the serum. This prospective study was performed on 63 suspicious cases of visceral leishmaniasis collected from the infectious diseases department at the Pediatric Hospital of Oran from January 2012 to July 2017. For each patient, the epidemiological parameters, and the clinical and biological data were collected. Bone marrow and blood samples were drawn from all cases. Bone marrow was performed to research amastigote forms of *Leishmania *and to identify the species by PCR-sequencing. Blood samples were used to detect anti-*Leishmania* antibodies as well as parasite DNA. Patients from the Western regions were mostly from rural areas. Sensitivity of RT-PCR from the bone marrow and from serum was 95.45% and 94.44%, respectively. The immunochromatography allowed the disease’s diagnosis for 11 cases whose myelogram did not confirm the presence of the amastigote forms of *Leishmania*. Immunochromatography was revealed to be a good technique for disease diagnosis regarding the strongly evocative clinical signs. The results also suggest the interest of the RT-PCR assay from patient serum as a non-invasive sample, in the detection of parasite DNA.

Leishmaniases are parasitic diseases caused by the multiplication of a protozoan parasite*, Leishmania* in the reticulo- endothelial system cells. They are transmitted by the bite of a vector insect, the female sandfly. Algeria is the most affected country in the Mediterranean basin and in the Maghreb by two clinical forms, cutaneous leishmaniasis and visceral leishmaniasis. Both are part of the notifiable diseases.

Visceral leishmaniasis is distributed on the northern part of the country at the bioclimatic humid and subhumid level floors, mainly in Kabilye. Currently, it extends into all areas; many cases are reported in the foci of zoonotic cutaneous leishmaniasis (1, 2).

Leishmaniasis usually affects young children with 111 new cases each year (3). The most often isolated parasite is *Leishmania infantum* zymodeme MON-1, having as the animal reservoir; the dog, and as vectors, *Phlebotomus perniciosus* and *P. longicuspis* (4, 5).

The epidemiological situation of IVL in Western Algeria is poorly documented. The diagnosis is still based on direct examination of the parasite in the bone marrow smear after staining.

The aim of this study was to draw up epidemiological, clinical and biological profile of IVL in Western Algeria through children hospitalized at the pediatric hospital of Oran, and to show the convenience of serum for visceral leishmaniasis diagnosis by PCR and by immuno-chromatography.

## Materials and methods

**Patient and sample collection**

This prospective study has concerned 63 suspicious cases of IVL collected from the infectious diseases department at the Pediatric Hospital of Oran over a 67 months period. The children were hospitalized under clinical and biological arguments, evoking a visceral leishmaniasis.

For each patient included in the study, the epidemiological parameters (age, sex, geographical origin, etc.), the clinical and biological data, were collected from the medical record. Bone marrow and blood samples were drawn from all cases. The anonymity of the patients and the confidentiality of their information were respected.

Bone marrow, collected at the level of the iliac crest, was spread in thin smear, on slides, fixed with methanol or the May Grünwald, and colored with Giemsa for the research of *Leishmania*’s amastigote forms. After reading, the slides were kept at room temperature.

The blood samples drawn in dry tube were centrifuged at 1509 xg for 5 min. After separation, 20 µl of sera were placed on the absorbent part on the strip of the rapid test by immunochromato-graphy for the detection of anti-*Leishmania *antibodies. The remaining sera were later aliquoted into Eppendorf tubes, and stored at- 20 C.

To evaluate its specificity, the immunochro-matography was carried out on 27 serum samples of patients with other pathologies (tuberculosis, malaria, toxoplasmosis, brucellosis, hepatitis B, cutaneous leishmaniasis, acquired immunodefi-ciency syndrome (AIDS), and polyarthritis), and 10 serum samples from negative controls.

A total of 22 bone marrow smears from confirmed cases were scraped using a sterile scalpel. The scraping powder of each of these smears was placed in a previously coded sterile Eppendorf tube. To evaluate its specificity, the bone marrows of 9 cases with other pathologies as well as that of a negative control case were introduced in the study.

The sera and powders of the conserved bone marrows were intended for molecular study by RT-PCR and PCR-sequencing.

The research protocol was approved by the Ethics Committee of University Hospital of Oran (EHU).

**DNA Extraction**

DNA was extracted from the two types of samples using Qiagen EZ1 kit (QIAamp DNA tissue kit, Qiagen, Germany). 200 µl of each patient's serum was deposited in a flat-bottom sample tube provided in the kit. The tube was the nplaced in the extraction automat (EZ1 Biorobot QIAGEN).

Pretreatment of bone marrow’s scraping powder by 200 µl of buffer and 20 µl of proteinase K, and a dry incubation at 56 **C** for 2 h was performed. The mixture was centrifuged at 3578 xg for 5 s, and 200 µl of the supernatant of each sample was placed in a flat bottom tube.

For both sample types, the program with 200 µl of elution volume was selected and launched.

**Real time PCR**

Parasites of the genus *Leishmania* have been targeted by RT-PCR in a CFX96 Real-time System (Bio-Rad, Marnes la Coquette, France) instrument, using the following primers: Leish F (5' ACAAGTGCTTTCCCATCG 3'), Leish R (5' CTCAGAGGCCGTGAGTTG 3') and the probe Leish P (6FAM CGGTTCGGTGTGTGGGSC-C). The final volume of the PCR mixture was 20 µl (15 µl Mix (Takyon)+5 µl DNA). Amplification was done as follows: an initial denaturation step at 95 C for 5 min, followed by 40 cycles of denaturation at 95 C for 30 s, annealing and elongation at 60 C for 60 s.

**PCR Amplification and sequencing**

Standard PCR instrument (AB Applied Biosystems, USA) was used to detect the *Leishmania* parasite by amplifying 500-, 400-and 800 bp segments of the internal transcribed spacer1 (ITS1) region, the internal transcribed spacer2 (ITS2) region, and the cytochrome b (Cytb) gene, respectively, using primers: ITS1 R (5' CAC GGGGATGACACAATAGAG 3'), ITS1 F (5' CAATACAGGTGATCGGACAGG 3'), ITS2 R (5' GGCCAACGCGAAGTTGAATTC 3'), ITS2 F (5' GCATGCCATATTCTCAGTGTC 3'), LCYT S (5' GGTGTAGGTTTTAGTYTAGG 3')] and LCYT R (5' CTACAATAAACA AATCATAATATRCAATT 3').

The final volume of the PCR reaction was 50 µl (45 µl Mix (ATQ Gold Master amplifier)+5 µl DNA). The technique was performed with an initial denaturation step at 95C for 15 min, followed by 40 cycles of denaturation at 95 C for 45 s, an annealing temperature which depends on the used primer (ITS1: 55 C, ITS2: 60 C; CYTB: at 52 C) for 30 s, and elongation at 72 C for 1 min, with final extension step at 72 C for 5 min.

The PCR products were analyzed using agarose gel electrophoresis and visualized in a darkroom on the UV table of the Doc XR+ gel transilluminator (BIO-RAD). Positive PCR products were then purified and sequenced in both directions using the BigDye® Terminator. Sequencing was performed on an automated ABI PRISM 3130 sequencer (Applied biosystems, Foster City, CA). The resulting sequences have been analyzed, corrected, and assembled using the CodonCode Aligner software.

*Leishmania* species were identified by using a BLAST (basic Local Alignment Search Tool) accessible in the molecular database (Genbank, NCBI) available at: https: // blast. ncbi. nlm. nih. gov/ Blast.cgi. DNA sequences from each parasite were retrieved in FASTA format and aligned with other sequences referenced in the GenBank. The similarity percentages were determined using the MEGA 7 software.

**Statistical analysis**

In this descriptive study, all parameters have been analyzed by the Epi Info software version 6.04d f. The average and standard deviation were calculated for the quantitative variables and the percentage for the qualitative variables.

Sensitivity, specificity, negative and positive predictive values were estimated with confidence intervals at 95%.

The Youden Index (j) and the coefficient of Yule (Q) were introduced for the first time in the evaluation of the rapid test’s performance by immunochromatography (rk39).

## Results

A total of 39 patients were retained in this study. The others (n=24) were excluded because other diseases with clinical signs that were similar with those of LV have been confirmed, revealing either malaria and leukemia.

**Fig. 1:**
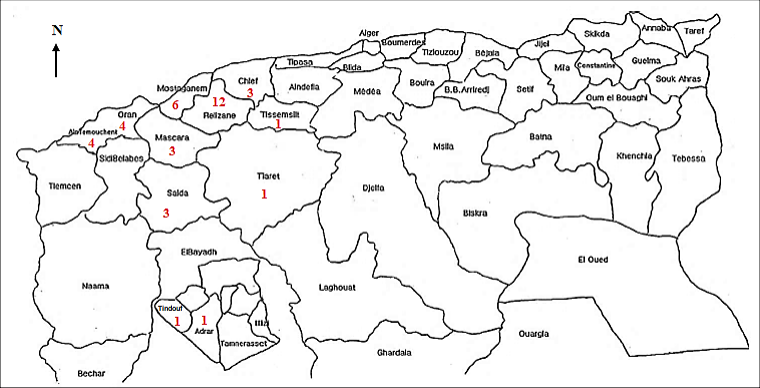
Distribution of cases according to the state of provenance

Patients were divided into 21 boys and 18 girls, with a sex ratio of 1.17. The average age was 2.16±2.48 years with extremes ranging from 7 months to 14.5 years; 94.87% of patients were less than 5 years old. All patients were from Western Algeria, Relizane (12), Mostaganeme (6), Oran (4), Ain Temouchent (4), Chlef (3), Mascara (3), Saida (3), Adrar (1), Tiarat (1), Tindouf (1) and Tissemsilt (1) (Figure 1).

89.74% (n=35) of patients originated from a rural or semi-urban environment, and 79.49% (n=31) had low socio-economic level. The disease occurred in Spring in 48.72% of cases (n=19).

The average hospitalization time of patients was 65.89± 66.03 days with extremes of 7 to 360 days, and the diagnosis period was 7.92±13.93 days with extremes from 1 to 60 days. The reasons for hospitalization are shown in table 1.

**Fig. 2:**
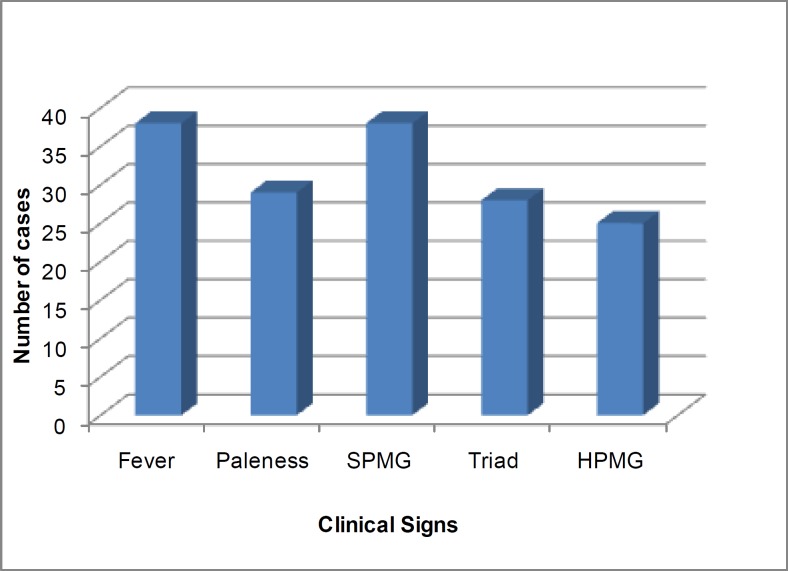
Distribution of cases according to clinical signs suggestive of the VL. HPMG : hepatomegaly; SPMG: splenomegaly

The evocative clinical signs of LV are shown in Figure 2; they are dominated by the classic triad: fever, paleness, and splenomegaly (SPMG).

The temperature was taken daily, every 3 h was intermittent, irregular with one to four peaks (39-42 C) interspersed of apyrexia periods.

**Fig. 3:**
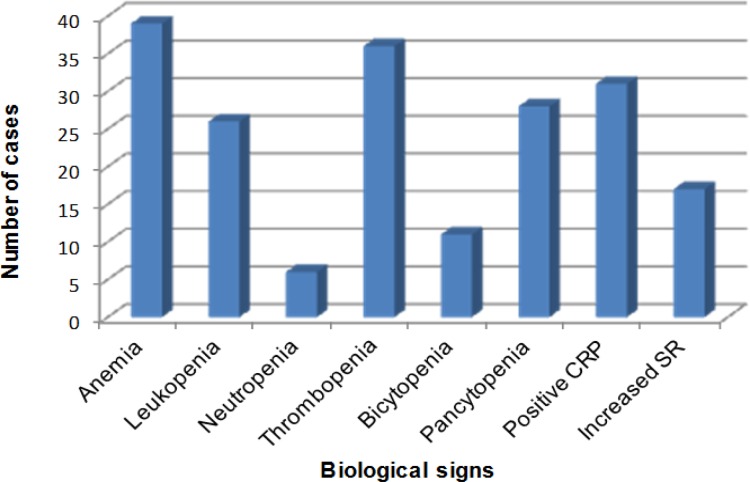
Distribution of cases according to the biological signs. SR: Increased sedimentation rate

Biological signs were represented in 100% of cases by anemia, mainly of hypochromic microcytic type with a hemoglobin rate ranging from 3.4 to 10.1 g/dl. It was corrected in 94.87% of cases by the blood transfusion. Leukopenia, thrombocytopenia, and C reactive protein (CRP) were observed in 66.67%, 92.31%, and 79.49% of cases respectively (Figure 3).

Apart from the clinical signs suggestive of the VL, the patients showed less frequent signs, represented in table 2.

Four cases have presented a macrophage activation syndrome (MAS) secondary to visceral leishmaniasis, in which the diagnosis was posed by the presence of fever, cytopenia and splenomegaly, associated with the biological parameters indicated in table 3.Direct exam and immunochromatography performed for all patients showed a positivity rate of 44, 44% and 57.14%, respectively.

**Fig. 4:**
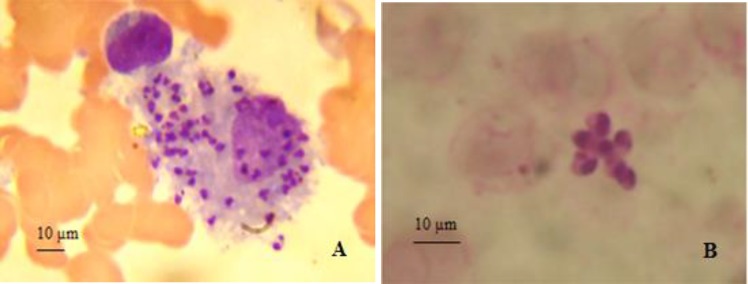
*Leishmania* amastigote forms. A: intracellular and B: extracellular forms observed under optical microscope (x 1000)

Myelogram showed the amastigote forms of *Leishmania* in 71.8% (n=28) of cases (Figure 4). The immunochromatography detected the anti-*Leishmania* antibodies in 92.3% (n=36) of cases. This latter showed a specificity and a positive predictive value (PPV) of 100%, a negative predictive value (NPV) of 88.9%, a Youden index (j) of 0.92 and a Yule's coefficient Q equivalent to1.

The absence of the test line on the immuno-chromatography strips in control cases sera justifies the lack of cross -reaction between VL and other infections (tuberculosis, malaria, toxoplas-mosis, brucellosis, hepatitis B, cutaneous leishma-niasis, AIDS and rheumatoid arthritis) and increases the specificity of the test.

RT-PCR, realized from the bone marrow’s scraping powder of the 22 confirmed cases, has detected parasitic DNA in 21 of them. It showed a sensitivity of 95.45%, and a specificity of 100%. RT-PCR made from sera of 33 patients, showed a sensitivity of 93.9%, and a specificity of 100%.

**Fig. 5:**
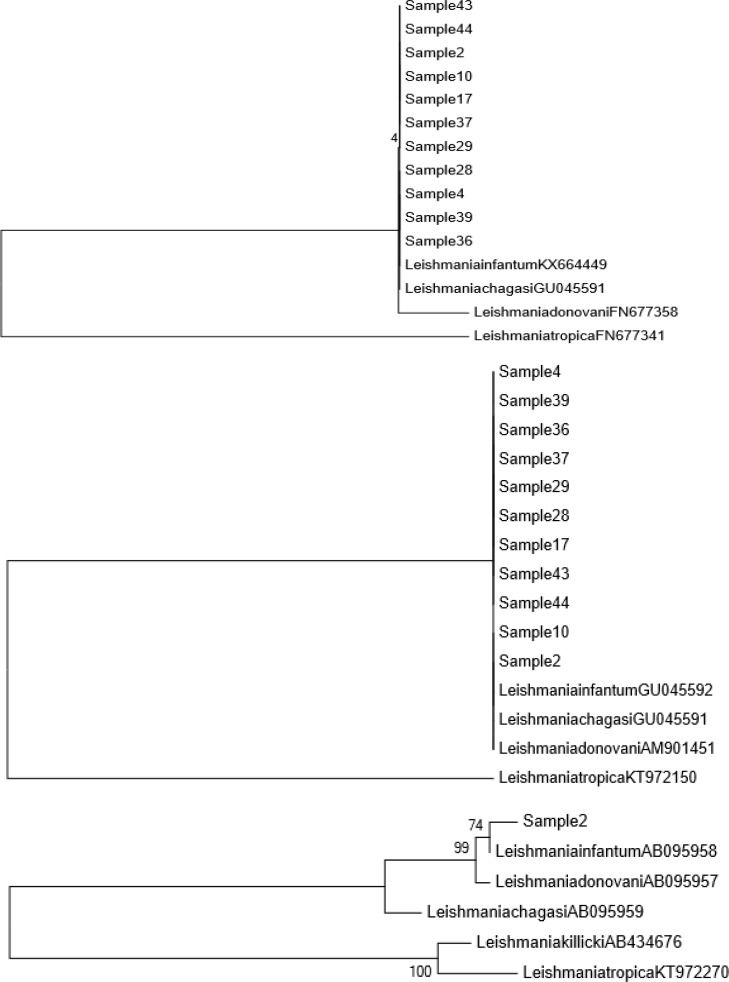
**Phylogenetic tree of the** ***Leishmania*** **species.** Phylogenetic tree was drawn based on sequences of the ITS1 (500 pb), ITS2 (400 bp), and Cytb (800 bp) amplified from bone marrow samples of prospective cases

Phylogenetic analysis revealed that the obtained sequences were regrouped and corres-ponded to the sequences of *L. infantum*. In the case of Cytb, the sequence assembly was only possible for one case (Figure 5).

**Table 1:** Hospitalization motifs of patients

**Hospitalization motif**	**Number of cases**	**Percentage**
Febrile hepatosplenomegaly	13	33,33
Fever	11	28, 2
Febrile splenomegaly	8	20,51
Febrile splenomegaly and Pancytopenia	3	7,69
Pancytopenia	1	2,56
Fever and Pancytopenia	1	2,56
Bicytopenia	1	2,56
Febrile hepatosplenomegaly and bicytopenia	1	2,56
Total	39	100

**Table 2:** Other clinical Signs

**Clinical signs **	**Number of cases**	**Percentage**
Anorexia	13	33,33
Asthenia	14	35,90
Cough	10	25,64
Altered general status	7	17,95
Vomiting	6	15,38
Malnutrition	6	15,38
Epistaxis	6	15,38
Edema	4	10,26
Oral candidiasis	5	12,82
Skin Lesions	3	7,69
Slimming	4	10,26
Diarrhea	1	2,56
Dehydration	2	5,13
Peri-rectum abscess	2	5,13
Cervical adenopathy	2	5,13

**Table 3:** Biological parameters in VL-MAS cases

Biological parameters	**Patient 1**	**Patient 2**	**Patient 3**	**Patient 4**
Leukocytes (4 000-10 000/mm^3^)	1230	2640	2900	3250
Hemoglobin (12-18g/dl)	4,7	4,6	3,4	3,4
Platelets (150 000-400 000/mm^3^)	37 000	1100	6000	9000
Ferritin (20-300µg/L)	**11 645,33**	NR	**70 786**	**96 820**
Triglycerides (<1,5nmol/L)	**1,56**	**3,6**	**4,94**	**4,3**
C-reactive protein (<10mg/L)	87,39	60	>60	36
Fibrinogen (2-4g/L)	3,37	**1,66**	3,74	**1,18**

NR: Not realized

Among the patients of our series, 37 cases were treated with N-methyl-glucamine (Glucantime ®), of which 36 were given two cures of 15 days each and 15 days of interval by deep intra muscular injection at the dose of 20 mg/Kg/day. The 37th who has presented a stibio-intolerance’s sign (fever resumption) that received a single cure of 30 days at the same dose. A case that healed spontaneously did not need the treatment. One patient died before receiving his treatment, and two others did not respond to the treatment.

## Discussion

In the Maghreb countries, IVL is predominant in young children in which 95% of cases are less than 5 years old, realizing the infantile Mediterranean Kala-azar (6). The disease in this age group has already been registered in Algeria (7, 8). The disease occurrence is explained by a beam of arguments among which the immaturity of the child's immune system and the sandfly's most marked affinity for this latter (9).

The appearance age of the disease corresponds to the period in which the infant loses maternal antibodies (7-15 months) and becomes sensitive to pathogens (10). However, cases were indicated in young adolescents aged from 13 to 14 years old (11). These results support those in our series.

A slight male dominance is noted in the majority of series (12), which reinforces our results. But according to the synthesis made on the VL by Carré and al. (2010), there is no gender difference outside the Indian hotbed where the prevalence of male subjects is due to their greater exposure to bites than to a particular sensitivity. 84.61% of the surveyed cases originated from the Algerian northwest regions, with humid or subhumid bioclimatic levels. The work of Harrat and collaborators (7, 13) emphasizes the likely emer-gence of new hotbeds particularly in this part of the country.

15.38% of the cases (n = 6) originated from the highlands and the Sahara, areas with arid or semi-arid climates, favorable to parasitic cycle of zoonotic cutaneous leishmaniasis (7).

Also, the majority of this child population was derived from a rural or semi-urban environment which combines the favorable conditions to the parasite’s transmission, the animal’s reservoirs (stray and domestic dogs) are numerous, and the larvae find in abundance the organic matter necessary for their development.

The high rate of cases in Spring (48.72%) would be related to the seasonal transmission of *L. Infantum* from July to October, which could also explain the appearance of the first symptoms in 28.57% of cases in March. The same observations are resulting from studies performed in Tunisia(14,15).

Prolonged hospitalization is probably due to the absence of rural population awareness campaigns on the severity of VL. Underestimation by parents of certain early symptoms (moderate non-continuous fever or discrete anemia) extends this period and, as a result, cases are registered throughout the year (10).

The case distribution of our study throughout the year and their late hospitalization has led to serious complications and even death.

As clinical signs, the majority of the patients had a splenomegaly, a skin-mucous pallor, and a fever that resisted antibiotic treatment. The triad: paler-splenomegaly-fever, signing in favor of an IVL, was observed in 71.79% of cases, and hepatomegaly was present in in 64.10% of cases. These results are close to those found in other studies (Table 4).

Macrophage activation syndrome secondary to visceral leishmaniasis (MAS-VL) is a predominantly pediatric disease as shown in the study of 56 cases including 8 adults only (20). It is due to an immune dysregulation favored by hyperproduction of cytokines by the infectious agent, *Leishmania*. The diagnosis of MAS was retained in 4 children by the presence of 5 characters out of 8 cited by Henter et al. (21).

The specific biological signs of IVL, found in the literature, correspond to a bicytopenia or a pancytopenia, and an important inflammatory syndrome (22, 12). These signs reinforce our observations with the presence of bicytopenia in 28.21% of cases, and pancytopenia in 71.79% of cases. The inflammatory syndrome was represented by a positive CRP in 79.49% of cases, and by accelerated erythrocyte sedimentation rate in 43.59% of cases.

**Table 4:** Rate of clinical signs appearance of the VL according to some literature studies

Studies	SPMG	Fever	Triad	HPMG	Paleness
(Oran, Algeria, 1^st^ study) (n=14) (16)	92,85%	100%	-	85,71%	-
(Fes, Morocco) (n=209) (12)	97.66%	94.5%	-	47.36%	50%
(Rabat, Morocco) (n=93) (17)	98,9%	92,4%	76,3%	48,4%	-
(Oran, Algeria, 2^nd^ study) (n=40) (18)	90%	87.5%	47.5%	42.5%	60%
(Kairouan, Tunisia) (n=240) (19)	97,9%	79,9%	-	47,3%	-
Our study (n=39)	97,44%	97,44%	71,79%	64,10%	74,36%

SPMG: splenomegaly; HPMG: hepatomegaly.

**Table 5:** Summary of the bibliographic data

**Studies**	**Se**	**Sp**	**PPV**	**NPV**	**J**	**Q**	**Serum number**
Italia (28)	100%	100%	-	-	-	-	11
Bresilia (1^st^ study) (30)	85.7%	82%	-	-	-	-	21
Nepal (1^st^ study) (31)	97%	71%	-	-	-	-	139
Nepal (2^nd^ study) (32)	100%	100%	-	-	-	-	14
India (33)	99%	89%	-	-	-	-	150
French (34)	97%	98%	89%	94%	-	-	34
Bresil (2^nd^ study) (35)	93%	97%	98%	89%	-	-	332
Tunisia (36)	87.1%	94.4%	96.2%	81.9%	-	-	574
Present study	92,3%	100%	100%	88,9%	0,92	1	100

Se: sensitivity; Sp: specificity; PPV: positive predictive value; NPV: negative predictive value; J: Youden index; Q: Yule's coefficient.

A positive CRP associated with other signs suggestive of VL, is a good element of diagnosis orientation. This diagnosis is often difficult in front of non-specific clinical signs that are confused with those of other infections (malaria, tuberculosis, brucellosis, enteric fever,..) (23, 24). The direct examination of the bone marrow, thus represents the reference method, and of the first intention of the parasitosis's diagnosis. The literature reports a positivity rate of 54 to 85% which may even exceed 90% when the examiner is experienced (25, 26). In this study, the diagnosis of the disease was brought by the direct exam in 66.67% of the cases.

This exam was negative in 11 cases, probably due to the hemodilution of the sample and the very low parasitaemia.

*Leishmania* species usually induce a cellular and humoral immune response with high antibody production. Their search in the host’s serum is therefore recommended.

The rapid diagnosis test by immunochromat-ography (RK39) has highlighted anti-*Leishmania *antibodies in 92.3% of cases (n=36), that joins the literature data, which also reported a sensitivity of 53.3% to 100% (27, 28). It has allowed the disease diagnosis in 11 patients for whom the direct exam was negative. The performance of this test (sensitivity, specificity, PPV and NPV) was relatively similar to those described in the literature (Table 5).

Front of the clinical and biological signs suggestive of the VL, the index of Youden of (0.92) (result close to 1) and the coefficient Q of Yule equivalent to 1, increase the performance of the rk39 in the diagnosis of the disease, especially in the developing countries.

The rk39 is a very interesting test, but it gives false negative results in the early stage of the disease, and does not distinguish a recent infection from an old one, thus limiting its use for relapse diagnosis (29).

Recently, RT-PCR has been successfully used for the diagnosis of leishmaniasis (30, 15) because of its sensitivity and specificity (31).

In this study, the sensitivity of 95.45% and the specificity of 100%, place the real-time PCR made from bone marrow scraping powder at peak of the list of VL diagnosis techniques. It can therefore be used as a routine diagnosis method. PCR-Sequencing presented an interesting alternative to conventional methods for the identification of *Leishmania*’s species. It has highlighted the presence of *L. infantum* in the Western Algeria through the cases studied.

The RT-PCR performance in the VL diagnosis was evaluated from serum samples of 33 patients; The sensitivity was 93.9%. Fissore et al., and De Assis et al. reported a sensitivity of 97% and 85%, respectively (32, 33).

The absence of DNA amplification of serum samples from two patients in our series could be explained by the fact that *Leishmania*, an intracellular parasite, was just going into the serum.

Due to its sensitivity and simplicity, RT-PCR from serum samples represents a valuable tool for the diagnosis of visceral leishmaniasis and could even be used in epidemiological investigations to detect asymptomatic carriers.

In Algeria, the classical treatment of VL is based on the pentavalent derivatives of antimony, (Glucantime ®). It is usually administered on a 30-day treatment (28 days according to WHO recommendations) at the dose of 20 mg/kg/day. A single cure does not always lead to healing and must be repeated after a resting time (34). If left untreated, the IVL usually evolves towards death within a few months.

The evolution of treatment was favorable in 94.59% of cases (35/37). The persistence of fever (a sign of stibio-intolerance) in one child, required a change from two-cure regimen of 15 days into a single cure of 30-days. Because of the altered general condition followed by the cumulative hospitalization period, three deaths were deplored.

The results of this study suggest the benefit of a diagnosis by RT-PCR that can be made from a non-invasive sample; such as the patient's serum. The use of RT-PCR from bone marrow is recommended for highly suspected cases, with negative results. In front of evocative bio-clinical signs, the positivity of serology by immuno-chromatography allows a very strong presumption. Obtaining the specific profile by a confirmation technique such as western blotting may help to remove doubts about false negatives.

## Conflict of Interest

Authors declare no conflict of interest.
